# *Bacillus amyloliquefaciens-9* as an Alternative Approach to Cure Diarrhea in Saanen Kids

**DOI:** 10.3390/ani11030592

**Published:** 2021-02-24

**Authors:** Wenying Zhang, Huijie Xin, Nannan Jiang, Zhengbing Lv, Jianhong Shu, Hengbo Shi

**Affiliations:** 1 Zhejiang Provincial Key Laboratory of Silkworm Bioreactor and Biomedicine, College of Life Sciences and Medicine, Zhejiang Sci-Tech University, Hangzhou 310018, China; zhangwenying@mails.zstu.edu.cn (W.Z.); xinhuijie@mails.zstu.edu.cn (H.X.); jiangnannan@mails.zstu.edu.cn (N.J.); zhengbingl@zstu.edu.cn (Z.L.); shujianhong@zstu.edu.cn (J.S.); 2 College of Animal Science, Zhejiang University, Hangzhou 310058, China; 3 Key Laboratory of Molecular Animal Nutrition, Zhejiang University, Hangzhou 310058, China

**Keywords:** intestinal health, diarrhea, cytokines, microbial community

## Abstract

**Simple Summary:**

Diarrhea is often the main cause of neonatal deaths on dairy goat farms. Poor management leads to infection with pathogenic bacteria and viruses causing enteritis, the main cause of diarrhea. Efficient drugs should be developed to prevent and treat kids with diarrhea. In the current study, the role of GBacillus-9 in the prevention and treatment of Saanen kids suffering diarrhea was assessed. Our data showed that GBacillus-9 restored the intestinal microbial disorder that resulted from diarrhea and suggested that GBacillus-9 could be used as a product to improve the gut health of kids.

**Abstract:**

*Bacillus amyloliquefaciens-9* (GBacillus-9), derived from the intestinal tract of the white-spotted bamboo shark, secretes a variety of antimicrobial compounds that inhibit the growth of pathogenic bacteria. In this study, the role of GBacillus-9 in the prevention and treatment of Saanen kids with diarrhea was assessed. Six healthy kids (HL) and six kids with diarrhea (DL) were selected. All kids were fed with 0.3% (*w*/*v*) GBacillus-9 (spray power) in raw milk for two weeks. The proportion of kids with diarrhea decreased gradually as the trial progressed, and 100% DL kids were cured at day 15. GBacillus-9 increased the serum immunoglobulin (Ig) G, interleukin (IL)-4, and IL-6 concentration (*p* < 0.05). The amplicon sequencing analysis of the fecal bacterial community revealed that the fecal microbiota was remarkably different between the HL and the DL groups at day 0. After two weeks of feeding with GBacillus-9, no significant difference in fecal microbiota was observed between HL and DL groups at the phylum level. GBacillus-9 restored the intestinal microbial disorder associated with serum immunoglobulin and interleukin concentration. Correlation analysis showed that GBacillus-9 altered globulin and interleukin concentration and that immunoglobulin was associated with *Firmicutes*. Collectively, our results revealed that GBacillus-9 improved the gut health of kids by improving microbial homeostasis.

## 1. Introduction

Diarrhea is often the main cause of neonatal deaths on dairy goat farms. Poor management leads to infection with pathogenic bacteria and viruses causing enteritis, the main cause of diarrhea [1]. Diarrhea causes poor feed efficiency and reduces the animal’s resistance to other pathogens such as pneumonia [2]. Since kids are fed milk by bottle, scrupulous sanitation is required to prevent introduction of pathogens into the milk and kid, causing enteritis and diarrhea. Diarrhea can be caused by rotavirus, coronavirus, *E. coli*, *Yersinia* spp., *Giardia*, *Campylobacter jejuni*, and many other organisms, making prevention by the use of drugs difficult [3,4]. 

Commonly, infectious diseases are cured with the administration of antibiotics. However, the irrational use of antibiotics may cause drug-specific adverse effects, multidrug-resistant bacteria, and environmental pollution [5]. Evidence suggests that probiotics work in the animal intestines [5], which may be a promising strategy to treat or prevent kid diarrhea. Probiotics colonize the host intestines, promoting resistance to infections, improving host immune system differentiation and synthesis of nutrients [6]. Probiotics participate in the immune response modulation by increasing the macrophage activation [7] and altering the release of proinflammatory and anti-inflammatory cytokines [8,9,10,11]. Thus, the use of probiotics is considered a promising strategy for the prevention and the control of various infectious diseases [12]. 

Research in other species supports the use of *Bacillus amyloliquefaciens*, a Gram-positive bacterium as a probiotic. Through secreting a complex metabolite, *B. amyloliquefaciens* exhibits a broad-spectrum antibacterial activity [13,14]. Data in chicks show that offering *B. amyloliquefaciens* changes the fecal metabolites involved in amino acid metabolism and the glyceride metabolism by altering the intestinal microbiota [15]. *B. amyloliquefaciens-9* (GBacillus-9 or Bacillus sp. GFP-2), which secretes antibacterial compounds, such as β-1,3-1,4-glucanase and antimicrobial peptides, is isolated from the intestinal tract of the whitespotted bamboo shark *(Chiloscyllium plagiosum)* [16]. The in vivo data in the hybrid sturgeon (*Acipenser sinensis*) show that dietary GBacillus-9 improves immunity by increasing the content of lysozyme in the skin mucosa [17]. Based on its antimicrobial activity [18], dietary GBacillus-9 increase the survival rate and the nonspecific immune defense system of Japanese eel (*Anguilla japonica*). These data suggest that GBacillus-9 can be used as a potential “drug” of marine origin to help decrease the use of antibiotics in agriculture. However, information about the role of GBacillus-9 in the prevention and treatment of enteritis is limited.

Therefore, this study aims to assess GBacillus-9 as a treatment for kid diarrhea. Our data showed that GBacillus-9 works efficiently in treating the diarrhea of Saanen kids. The intestinal health of kids was improved and associated with the alteration of the immune system and the fecal microbiota. 

## 2. Materials and Methods 

### 2.1. Ethics Statement

This study was carried out following the regulations of Instructive Notions with Respect to Caring for Experimental Animals. The project (code: 201809006) was approved by the Experimental Animal Management Committee of the Zhejiang Sci-tech University. 

### 2.2. Preparation of GBacillus-9

The GBacillus-9 (CGMCC number: 13337, accession number: CP021011) was isolated from the intestinal tract of whitespotted bamboo shark (*C. plagiosum*) [16]. The fermentation of GBacillus-9 was prepared following the reported procedure [18]. The GBacillus-9 powder was prepared using a spray dryer (L-217, 1.0 mm nozzle, Lai Heng, Beijing, China). The inlet air temperature, aspirator, liquid flow, and compressed spray airflow were set at 55 °C, 2 L/h, and 50 L/h, respectively. Corn starch was used as an adhesion agent at ratio of 100 g/L. The colony-forming units in the powder were more than 2 × 10^9^ /g.

### 2.3. Animal Experimental Design

The diagnosis of diarrhea was made by a veterinarian according to the stool firmness [19]. A modified photographic Bristol stool form scale was used. The diarrhea status of kids was assessed and recorded by a veterinarian at day 0, 7, and 15. The stool hardness classifies feces from 1 to 5 according to the stool’s appearance, in which a score of 1–2 corresponds to normal stools (“pellet, clumpy, or log poop”), a score of 3 to intermediate, and a score of 4–5 to mushy consistency and completely liquid stools. The status of kid health was assessed with assessments three times in a day. The average score > 3 was defined as diarrhea. The detail of the kid and stool form is supported in the supporting file (Figure S1).

A total of 12 female Saanen dairy goat kids (age = 30 ± 2 days, initial bodyweight = 6.5 ± 1.05 kg, multiple parturitions) contained six healthy kids (HL) and six kids suffering diarrhea (DL). All kids consumed colostrum from their dams and nursed them for seven days before separation. Each kid was raised in individual nursery in a feeding room at 25 °C. Before the experiment, the floor and walls of the building were thoroughly disinfected in accordance with animal care protocol. No medical treatment for the DL kids was performed, but all the DL kids survived during the whole period. 

Kids were fed 0.3% (*w*/*v*) GBacillus-9 added to raw milk at 37 °C for two weeks. Kids were fed 500 mL fresh milk at 8 a.m. and 4 p.m. every day. All kids had access to complete formula, granulated feed, and alfalfa, and drank warm boiled-water ad libitum throughout the experiment.

### 2.4. Serum Collection and Biochemical Analysis

On day 15, 3 mL of blood was collected from the jugular vein of each kid at 9 a.m. to 10 a.m., and serum was separated by centrifugation. The serum factors related to oxidative damage (malondialdehyde (MDA) and superoxide dismutase (SOD)), and factors related to physiological status (glucose (Glu) and triacylglycerol (TG)) were analyzed. The immune factors in serum-containing immunoglobulin A (IgA), immunoglobulin G (IgG), immunoglobulin M (IgM), interleukin-2 (IL-2), interleukin-4 (IL-4), and interleukin-6 (IL-6) were also analyzed. These factors were measured using specific ELISA commercial kits (Nanjing Jiancheng Biotech, Jiangsu, China) and semiautomatic equipment (BioPlus 2000, Kolding, Denmark, Europe) [20,21].

### 2.5. Fecal Collection and 16S rRNA Gene Sequencing

To investigate the alteration of microbial community, six healthy kids and three kids with diarrhea (shared a stock ram with the kids in HL group) were selected for fecal collection at day 0. Consistently, the feces of selected kids excepted the kid suffering diarrhea (containing five kids from HL group and three kids from DL group) were also collected on day 15. The homogenized fecal sample was snap-frozen in liquid nitrogen and stored at −80 °C for the subsequent DNA analysis. The fecal sample was used for total genomic DNA extraction using a commercial kit (Tiangen Biotech, Beijing, China). The V3–V4 regions of the bacterial 16S rRNA genes were amplified and sequenced and paired-end sequenced (2 × 300 bp) on the Illumina MiSeq platform following standard protocols (Novogene Technology Co. LTD, Tianjing, China). 

### 2.6. Sequencing Data Analysis, Bacterial Richness, and Community Diversity

The process of raw reads of different samples was demultiplexed and quality-filtered following procedures previously described [22]. The Shannon, Chao1, and Simpson indices were used to estimate the bacterial richness and community diversity. 

### 2.7. Statistical Analysis

All statistical analyses were performed using the Student’s *t* test with the SPSS software (SPSS v.19, SPSS Inc., Chicago, IL, USA). Data were presented as mean ± SEM. *p* < 0.05 was considered statistically significant. Principal components analysis was applied to visualize the dissimilarity of microbial communities among different age groups. The Spearman correlation using the SPSS software was used to explore the relationships among immune indices and bacterial taxa. Significant correlations (*p* < 0.05) were displayed in the network. Correlation networks were generated using the Spearman’s rank correlation coefficients and visualized using the Cytoscape. The abundance of bacteria ≥ 0.0001 in more than 60% was used in the Spearman correlation analysis. The significant correlation between bacterial genus and the immune globulins and cytokines was considered when |R| > 0.2 and *p* < 0.05).

## 3. Results

### 3.1. Role of GBacillus-9 in the Prevention and Cure of Diarrhea in Kids

**Figure 1:**
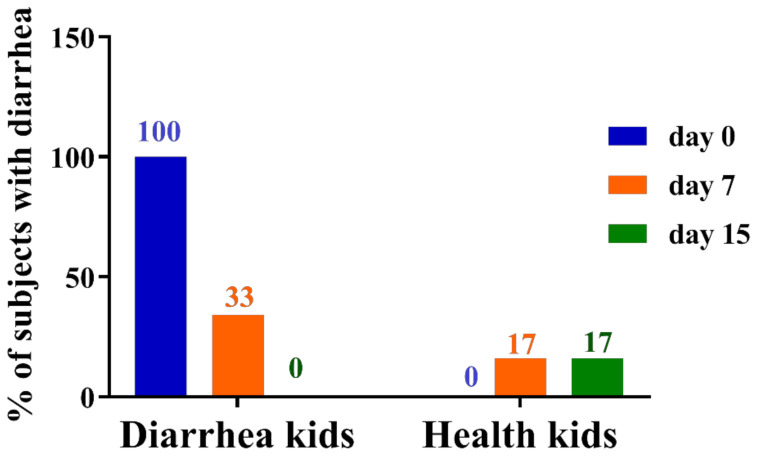
Effect of the administration of *Bacillus amyloliquefaciens-9* on the occurrence of diarrhea in kids with diarrhea (*n* = 6) and healthy kids (*n* = 6).

All kids were fed with GBacillus-9 for two weeks. The diarrhea score of kids was assessed and recorded by a veterinarian at day 0, 7, and 15. The ratio of kids suffering diarrhea decreased gradually during the experimental period (Figure 1). All kids with diarrhea (DL) were cured at day 15 (Figure 1), whereas only 17% of the initially healthy kids (HL) presented diarrhea at day 7 (Figure 1). 

### 3.2. Alteration of the Immune and the Biochemical Indices of DL Kids by GBacillus-9

**Figure 2:**
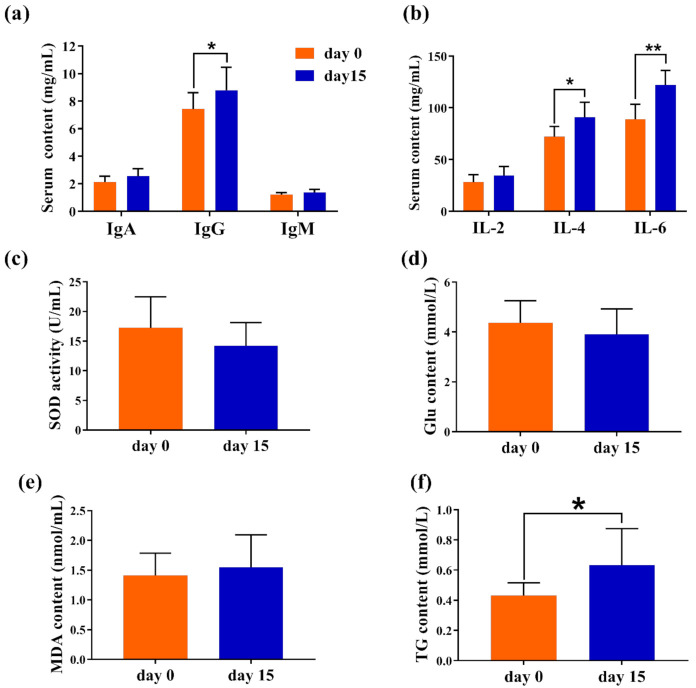
The effect of the administration of *Bacillus amyloliquefaciens-9* on serum immune and biochemical indices of six treated Saanen kids suffering diarrhea at day 0 and 15. Kids were fed with 0.3% (*w*/*v*) *Bacillus amyloliquefaciens-9* added to raw milk for two weeks. (**a**) IgA, IgG, and IgM concentrations. (**b**) Serum IL-2, IL-4, and IL-6 concentrations. (**c**) Superoxide dismutase concentration. (**d**) Serum concentrations of glucose. (**e**) Serum malondialdehyde concentration. (**f**) Serum triglyceride concentration. Data are means ± SEM. * *p* < 0.05 and ** *p* < 0.01 by Student’s *t* test.

The IgG concentration after GBacillus-9 feeding of DL kids was higher at day 15 compared to day 0 (*p* < 0.001, Figure 2a). Feeding GBacillus-9 significantly increased concentrations of interleukin (IL)-4 and IL-6 (*p* < 0.01, Figure 2b) of DL kids. No significant change was observed in IgA, IgM, and IL-2 concentrations (Figure 2a,b). No significant changes were observed in SOD, Glu, and MDA concentrations between day 0 and 15 (Figure 2c–e). A significant change in triglyceride (TG) concentration (*p* < 0.001) was observed between the day 0 and day 15 (Figure 2f). For the healthy kids, the feeding GBacillus-9 was similar to increase the concentration of IL-4 and TG (Supplementary Materials, Figure S2).

### 3.3. Comparison of the Fecal Microbiota between HL and DL Kids at Day 0

**Figure 3:**
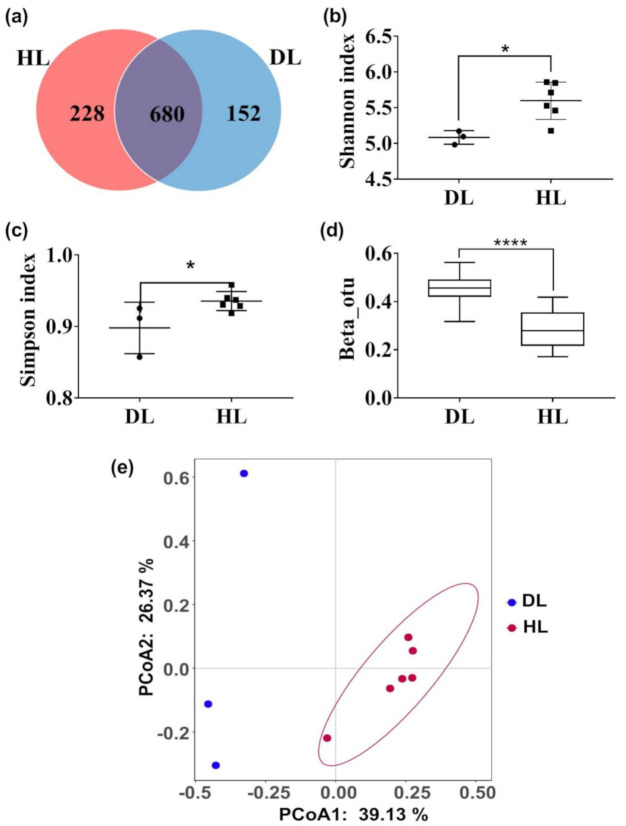
Fecal microbiota characteristics in three kids with diarrhea (DL) and six healthy kids (HL) collected at day 0. Bacterial 16S rRNA genes were amplified and sequenced. (**a**) OTU Venn Diagram, (**b**) Alpha diversity (Shannon index), (**c**) Alpha diversity (Simpson index), (**d**) Beta diversity, and (**e**) PCoA analysis (PCoA1 = 39.13%, PCoA2 = 26.37%) of the HL and the DL groups. Data are mean ± SEM. * *p* < 0.05, **** *p* < 0.0001.

The amplicon sequencing of the 16S rRNA gene was assigned to 1093 OTUs in all fecal microbiota. DL and the HL shared 680 OTUs and had specific 228 and 152 OTUs, respectively (Figure 3a). Fecal microbial diversities of HL and DL kids were analyzed. HL kids had higher Shannon and Simpson indices compared with DL kids (Figure 3b,c). DL kids had significantly higher Beta diversity than HL kids (*p* < 0.001, Figure 3d). Based on the health status, the principal coordinate analysis (PCoA) showed that samples clustered together (Figure 3e). This finding was also revealed by the similarity analysis (ANOSIM; R = 0.7284, *p* = 0.013), indicating a significant difference between groups.

At the phylum level, the *Firmicutes* and *Bacteroidetes* were the two dominant phyla, contributing 33.24% and 31.40%, respectively, in the DL kids and 65.34% and 18.44% in the HL kids, respectively (Table 1), followed by *Actinobacteria*, *Verrucomicrobia*, *Proteobacteria*, and *Cyanobacteria*. The abundance of *Firmicutes* in the DL kids was lower (*p* < 0.01) than that in the HL group. Significant differences were also observed in the levels of Proteobacteria and the *Fusobacteria* (*p* < 0.05, Table 1). The predominant taxa of bacteria at the genus level (relative abundance > 1%) consisted of 12 and 11 genera in HL and the DL groups, respectively (Supplementary Materials, Figure S3). However, no significant change was observed at the genus level (Supplementary Materials, Table S1).

### 3.4. Changes in the Fecal Microbiota between the HL and the DL Group after the GBacillus-9 Administration

**Figure 4:**
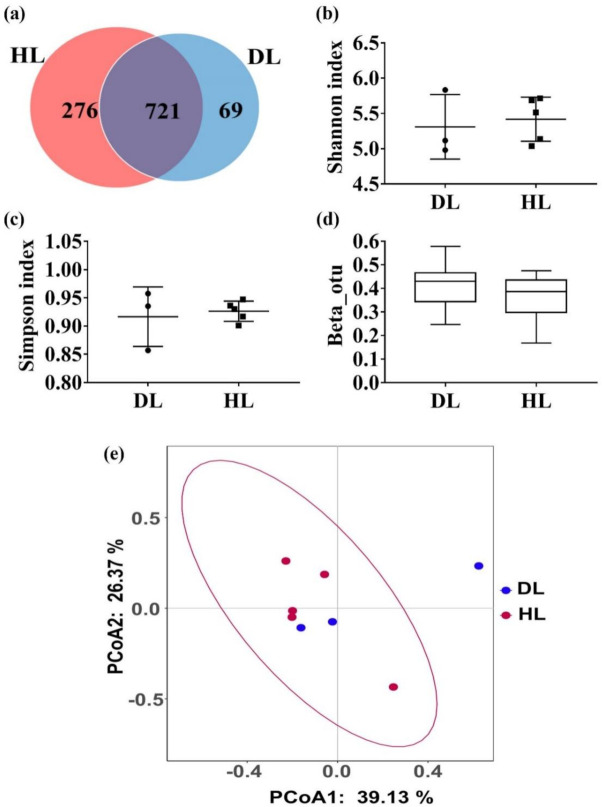
Fecal microbiota characteristics in three kids with diarrhea (DL) and five healthy kids (HL) after *Bacillus amyl-liquefaciens-9* administration collected at day 15. Bacterial 16S rRNA genes were amplified and sequenced. (**a**) OUT Venn Diagram, (**b**) Alpha diversity (Shannon index), (**c**) Alpha diversity (Simpson index), (**d**) Beta diversity, and (**e**) PCoA (PCoA1 = 39.13%, PCoA2 = 26.37%) of the HL and DL kids. Values are mean ± SEM. * *p* < 0.05 was considered statistically significant.

DL kids cured of diarrhea and HL animals were fed with GBacillus-9 for two weeks, and their feces were collected to assess changes in the fecal microbiota. The amplicon sequencing of the 16S rRNA gene was assigned to 1088 OTUs. DL and HL kids shared 721 OTUs and had specific 276 and 69 OTUs, respectively (Figure 4a). There was no significant change in the Shannon and the Simpson diversity indices between HL and DL kids (Figure 4b,c). The similarity of the microbial composition between the HL and DL was evidenced by the absence of significant change in the Beta diversity (Figure 4d). The PCoA with the Weighted-Unifrac analysis showed that the genetic structure composition of the DL kids was similar to that of the HL kids (Figure 4e), which was further confirmed by the ANOSIM (*R* = 0.006, *p* = 0.526).

At the phylum level, no significant difference was observed in all predominant microbiota (Supplementary Materials, Figure S4). The predominant bacterial group (Table 2) at the genus level (relative abundance > 1%) except *Erysipelotrichaceae_UCG-003* also showed no significant change (Supplementary Materials, Table S2).

### 3.5. Correlation between the Immune and the Dominant Genera of Fecal Bacteria

**Figure 5:**
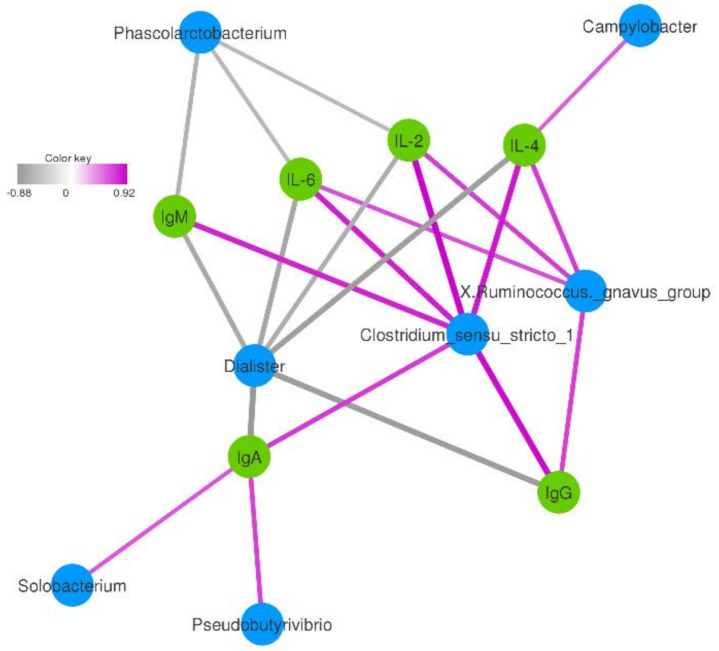
Correlation network showing the associations among lamb immunoglobulins, cytokines, and intestinal bacteria (relative abundance ≥ 0.1% in at least 60% of all samples). Only strong correlations (|*R*| ≥ 0.5 and *p* < 0.05) are displayed in the network. The edge width and the color (red: positive; grey: negative) are proportional to the correlation strength.

Further Spearman correlation analysis was performed to detect whether and how the dominant genera of fecal bacteria could be attributed to an increase in immunoglobulins, and cytokines, which help cure diarrhea. Data showed that seven genera, including *Campylobacter*, *Clostridium_sensu_stricto_1*, *X.Ruminococcus._gnavus_group*, *Dialister*, *Phascolarctobacterium*, *Solobacterium,* and *Pseudobutyrivibrio*, were correlated with immunoglobulins and cytokines (Figure 5). In the network, three genera were correlated with IgG. IgG was positively correlated with *Clostridium_sensu_stricto_1* (*R* = 0.89, *p* < 0.05) and *X.Ruminococcus. gnavus_group* (*R* = 0.69, *p* < 0.05) and negatively correlated with *Dialister* (*R* = −0.85, *p* < 0.05). *Clostridium_sensu_stricto_1* was strongly positively correlated with IgA, IgG, IgM, IL-2, IL-4, and IL-6. *Dialister* was negatively correlated with IgA, IgG, IgM, IL-2, IL-4, and IL-6. *X. Ruminococcus._gnavus_group* was positively correlated with IL-2 (*R* = 0.70, *p* < 0.05), IL-4 (*R* = 0.71, *p* < 0.05), and IL-6 (*R* = 0.64, *p* < 0.05). Except for *Campylobacter,* which belonged to the *Proteobacteria* phylum, all seven genera in the network belonged to the *Firmicutes* phylum. This result was consistent with the phylum change in kids fed with GBacillus-9.

## 4. Discussion

A limitation of the current study is the lack of an analysis of the possible diarrhea-by-treatment interactions, although evidence has shown the interaction of host immune and diarrhea in humans and animals [23,24,25]. In the present study, it was well established that probiotics interacted with the host by modulating gut macrobiotic composition, altering nutrient metabolism in the intestine, or directly improving intestinal barrier function [26,27]. Recent data suggested a role of probiotic *Bacillus* in pathogen elimination via signaling interference [28]. Probiotics are also suggested as antibiotic alternatives for the cure of human and animal diarrhea [29,30]. *Bifidobacterium lactis HN019* is reported to enhance immune protection by relieving the severe diarrhea of weaned piglets [29]. After the oral administration, the probiotic *Faecalibacterium prausnitzii* strain stimulates the secretion of IL-10 and reduces the FN-γ and IL-12 to treat calf diarrhea [30]. These data suggest that probiotics improve the immune system of the host [31]. However, there are few studies on the interaction mechanism between probiotics and hosts of large mammals like lambs. In the current study data suggest that the GBacillus-9 may be used as an “antibiotic alternative” to cure diarrhea in kids by altering the diversity of the intestinal microflora. 

Recent data show that administration of GBacillus-9 improves the immunity of the hybrid sturgeon [17]. The low percentage of kids with diarrhea in the group fed GBacillus-9 is consistent with findings showing that dietary GBacillus-9 improves the survival of fish [17]. Serum immunoglobulins play a role in resisting the invasion of bacteria and viruses, and their concentration can reflect the level of the organism’s resistance to exogenous pathogens [32,33]. The remarkable increase in IgG after GBacillus-9 feeding agrees with data in pigs infected with epidemic diarrhea virus [34]. The lack of change in IgM may be due to the increase in IgM, which often occurs at the early stage of the immune response. However, these data suggest the role of GBacillus-9 in the improvement of immunity in kids through the secretion of immunoglobulins. 

ILs play a key role in transmitting information and activating and regulating immune cells [35]. IL-6 stimulates the differentiation and maturation of B lymphocytes. The evidence that diet supplemented with EM Bokashi increases the concentrations of IL-2, IL-6, and IL-4 [36] is consistent with the present data in young kids fed with GBacillus-9. The increase in IL-4 and IL-6 after feeding with GBacillus-9 suggests the potential role of GBacillus-9 in increasing lamb immunity, which is further confirmed by the increase in IgG. 

The weak change in biochemical indicators (e.g., SOD and MDA) in blood suggests that GBacillus-9 does not alter the homeostasis of kids but specifically promotes disease resistance by increasing the IgG activity. The finding that the level of TG was higher after feeding with GBacillus-9 is consistent with previous reports in lambs [1]. These results suggest that GBacillus-9 increases the immunoglobulin activity and improves intestinal health.

The fecal microbiota plays an important role in resisting pathogenic infections [37], which are closely related to the occurrence of diarrhea [38]. In the current study, the fecal microbial diversity and abundance of the DL and the HL groups were remarkably different, indicating the correlation between the fecal microbial diversity and diarrhea. The difference in the diversities between HL and DL animals was ample before GBacillus-9 feeding and not significant after GBacillus-9 feeding, suggesting the role of GBacillus-9 in altering the fecal microbial diversity and further altered intestinal microbial diversity. These results were further confirmed with the PCoA and ANOSIM result. These data point to prevention of diarrhea with the administration of GBacillus-9 by altering the intestinal microbial community of the healthy group.

*Firmicutes* dominate the animal intestinal microbial community [39, 40, 41] and play an important role in degrading fiber and cellulose [42]. Notably, in this study, *Firmicutes* were the major phylum of fecal microorganisms in both the DL and the HL kids. The high abundance of *Firmicutes* in the HL group suggests that *Firmicutes* are an important microbial community that affects intestinal homeostatic functions. Combined with the finding that the abundances of *Firmicutes* in the DL and the HL groups after the GBacillus-9 feeding are not significantly different, our data reveal that GBacillus-9 promotes the microbial community of the DL group close to the HL group. The cure for kids suffering diarrhea was effective with GBacillus-9 by reverting the intestinal microbial disorder.

In this study, remarkable linkages between the immune indices and the abundance of intestinal bacteria were identified after GBacillus-9 feeding. Positive relationships were found between the five genera of bacteria and various immune indices. For instance, the levels of *Clostridium sensu stricto* 1 is high in healthy piglets and low in piglets with diarrhea [43]. The positive relationship between the five genera of bacteria and the immune indices in the current study suggests that some species belonging to these genera may positively increase the secretion of immunoglobulin or interleukin, and this idea needs further validation by using the isolates from this genus. The negative relationship between the concentration of immunoglobulin or interleukin and *Dialister* is consistent with the fact that *Dialister* is a microbial marker of disease activity in spondyloarthritis [44]. Of the seven genera of bacteria identified, six were correlated with the immunoglobulin or interleukin and belong to the *Firmicutes*, confirming the view that *Firmicutes* are an important microbial community affecting the intestinal homeostatic functions. A limitation of the statistics of was that the diarrhea was not considered as a fixed factor in the current study. It might influence the microbiota community and the parameters with some kids developing diarrhea during the study. However, these data still show the potential contribution of GBacillus-9 in the balanced physiological homeostasis of the goat kid intestine. 

In conclusion, these results demonstrated the role of GBacillus-9 in the treatment and prevention of goat kid diarrhea. GBacillus-9 feeding remarkably upregulates the concentrations of IgG, IL-4, and IL-6. Data indicating that the disorder of the microbial diversity is stabilized in kids with diarrhea by GBacillus-9 underscore the role of the GBacillus-9 in manipulating the microbial homeostasis in the kid intestine. The strong correlation between immunoglobulins or interleukins and seven genera of bacteria suggests that the GBacillus-9 strengthens the host immunity by altering the microbial homeostasis, thereby benefiting intestinal health. Further chemical studies to identify the compounds of GBacillus-9 responsible for the improvement of the health of kids with diarrhea fed this probiotic are needed. 

## Figures and Tables

**Table 1:** Comparison of top 10 phyla in the feces of kids with diarrhea (DL, *n* = 3) and healthy kids (HL, *n* = 6) at day 0.

Taxonomy	DL (%)	HL (%)	*p* Value
*Firmicutes*	33.24 ± 5.90	65.34 ± 9.16	0.002
*Bacteroidetes*	31.40 ± 12.16	18.44 ± 10.55	0.19
*Actinobacteria*	14.24 ± 18.50	9.59 ± 8.55	0.66
*Verrucomicrobia*	8.36 ± 8.13	0.26 ± 0.13	0.07
*Cyanobacteria*	4.31 ± 5.95	0.68 ± 0.66	0.23
*Proteobacteria*	7.82 ± 2.16	3.52 ± 1.18	0.01
*Euryarchaeota*	0.23 ± 0.11	1.74 ± 1.48	0.16
*Tenericutes*	0.17 ± 0.01	0.39 ± 0.48	0.50
*Fusobacteria*	0.15 ± 0.12	0.003 ± 0.003	0.028
*Saccharibacteria*	0.040 ± 0.037	0.037 ± 0.040	0.92

Significance was declared when *p* < 0.05 by Student’s t test.

**Table 2:** Comparison of top 10 phyla in the feces of kids suffering diarrhea (DL, *n* = 3) and healthy kids (HL, *n* = 5) fed with *Bacillus amyloliquefaciens-9* at day 15.

Taxonomy	DL (%)	HL (%)	*p* Value
*Firmicutes*	36.54 ± 8.36	49.47 ± 19.84	0.37
*Bacteroidetes*	37.05 ± 15.90	20.16 ± 7.28	0.095
*Actinobacteria*	16.40 ± 16.96	9.75 ± 8.59	0.51
*Proteobacteria*	6.31 ± 2.13	10.72 ± 12.77	0.62
*Verrucomicrobia*	2.17 ± 2.17	5.45 ± 7.68	0.54
*Cyanobacteria*	0.75 ± 0.91	2.84 ± 4.45	0.50
*Euryarchaeota*	0.39 ± 0.27	1.15 ± 0.97	0.28
*Tenericutes*	0.19 ± 0.06	0.31 ± 0.27	0.54
*Saccharibacteria*	0.06 ± 0.04	0.06 ± 0.08	0.97
*Fusobacteria*	0.12 ± 0.08	0.05 ± 0.07	0.32

Significance was declared when *p* < 0.05 by Student’s *t* test.

## Data Availability

Identified sequences were deposited in the NCBI Sequence Read Archive under the accession no. PRJNA666542.

## Supplementary Materials

The following are available online at https://www.mdpi.com/2076-2615/11/3/592/s1: Figure S1. A modified photographic Bristol stool form scale. The stool hardness which is classified from 1 to 5 according to the stool’s appearance, in which score 1–2 corresponds to normal stools ("pellet, clumpy or log poop"), score 3 to intermediate, score 4–5 to mushy consistency and completely liquid stools. The status of kid health was assessed as least triplicate. The average score >3 was defined as diarrhea; Figure S2. The effect of the administration of Bacillus amyloliquefaciens-9 on serum immune and biochemical indices of 6 healthy Saanen kids at day 0 and 15. Kids were fed with 0.3% (*w*/*v*) Bacillus amyloliquefaciens-9 added to raw milk for two weeks. (a) IgA, IgG, and IgM concentrations. (b) Serum IL-2, IL-4, and IL-6 concentrations. (c) Superoxide dismutase concentration. (d) Serum concentrations of glucose. (e) Serum malondialdehyde concentration. (f) Serum triglyceride concentration. Data are means ± SEM. * *p* < 0.05 and ** *p* < 0.01 by Student’s t test; Figure S3. The relative abundance analysis at phylum level of HL and DL’s intestinal microorganism in normal. The fecal simple from 6 health kids (HL) and 3 diarrhea kids (DL) were collected at day 0. Bacterial 16S rRNA genes were amplified and sequenced; Figure S4. The relative abundance analysis at phylum level of HL and DL’s intestinal microorganism treated with GBacillus-9. The fecal simple from 5 health kids (HL) and 3 diarrhea kids (DL) were collected at day 15. Bacterial 16S rRNA genes were amplified and sequenced; Table S1. The 20 most predominant genus in the feces of kids with diarrhea (DL) and healthy kids (HL) at day 0; Table S2. Comparison of top 20 genus in the feces of the DLs and the HLs fed with Bacillus amyloliquefaciens-9 at day 15.
