# Supplementary material for: Bacillus amyloliquefaciens-9 as an Alternative Approach to Cure Diarrhea in Saanen Kids
Source: Animals (Basel). 2021 Feb 24;11(3):592. doi: 10.3390/ani11030592 (PMC7996169; doi:10.3390/ani11030592)
Supplement: Supplementary file 1 [file animals-11-00592-s001.pdf]

Supporting information

The Goat Stool Chart			
**Classifications**	**Figure**	**Stool's Appearance**	**Condition**
**Type 1**	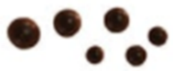	**Pellet poop**	**Normal**
**Type 2**	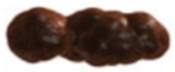	**Clumpy or log poop**	**Normal**
**Type 3**	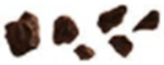	**Soft blobs with clear-cut edges (easy to pass)**	**Intermediate**
**Type 4**	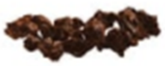	**Mushy consistency with ragged edges**	**Mild Diarrhea**
**Type 5**	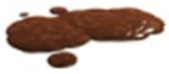	**Entirely liquid consistency with no solid pieces**	**Severe Diarrhea**

Figure S1 A modified photographic Bristol stool form scale. The stool hardness which is classified from 1 to 5 according to the stool's appearance, in which score 1-2 corresponds to normal stools ("pellet, clumpy or log poop"), score 3 to intermediate, score 4-5 to mushy consistency and completely liquid stools. The status of kid health was assessed as least triplicate. The average score >3 was defined as diarrhea.

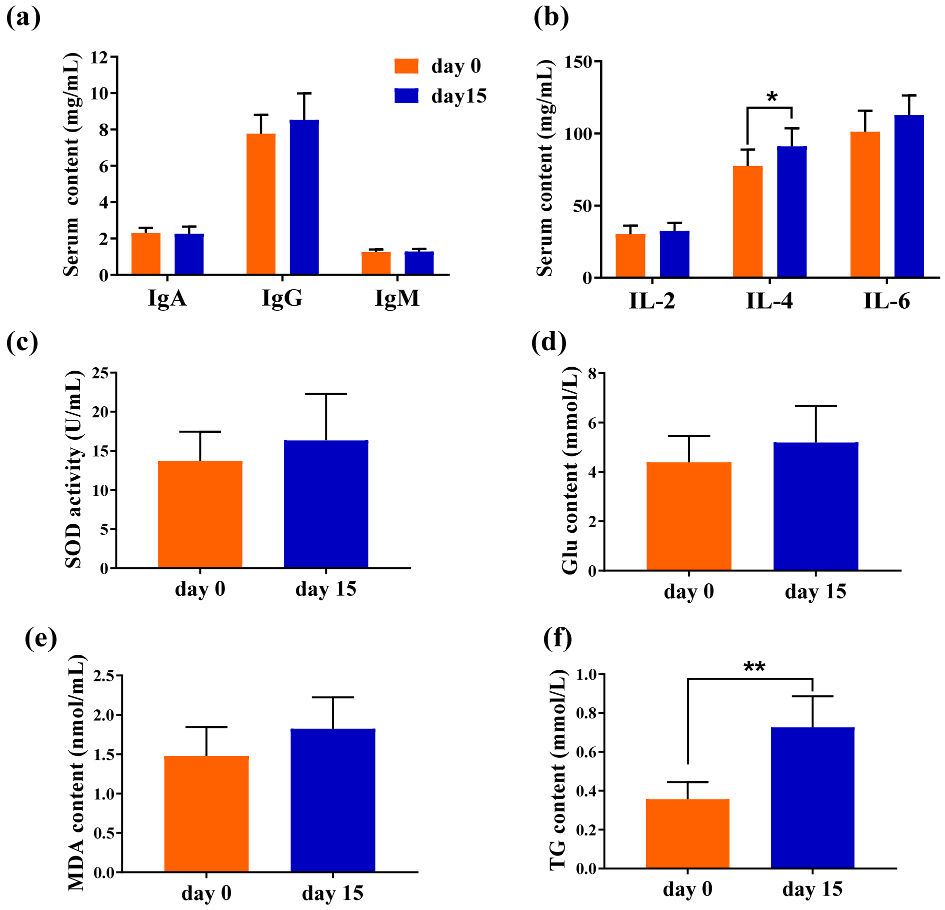

Figure S2. The effect of the administration of Bacillus amyloliquefaciens-9 on serum immune and biochemical indices of 6 healthy Saanen kids at day 0 and 15. Kids were fed with 0.3% (w/v) Bacillus amyloliquefaciens-9 added to raw milk for two weeks. (a) IgA, IgG, and IgM concentrations. (b) Serum IL-2, IL-4, and IL-6 concentrations. (c) Superoxide dismutase concentration. (d) Serum concentrations of glucose. (e) Serum malondialdehyde concentration. (f) Serum triglyceride concentration. Data are means ± SEM. *$p < 0.05$ and **$p < 0.01$ by Student's t test.

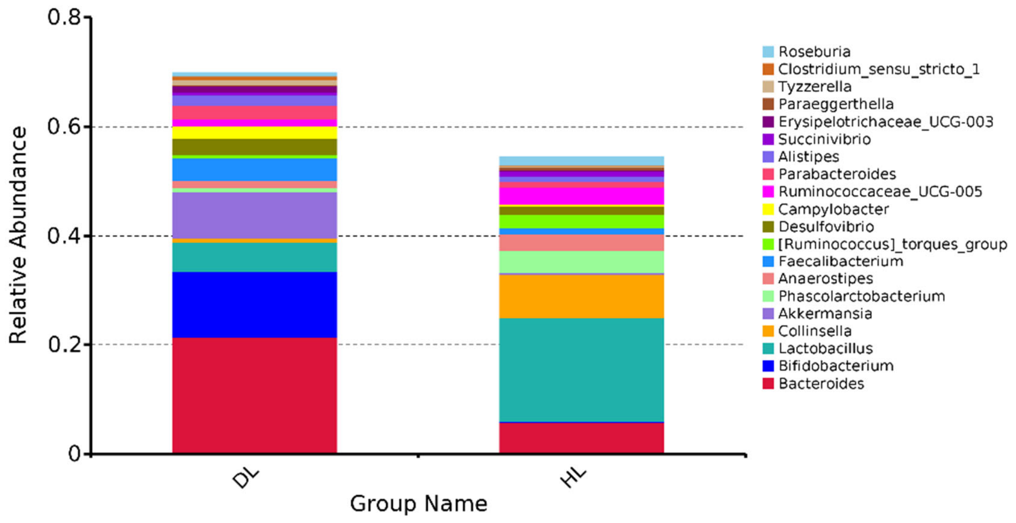

Figure S3 The relative abundance analysis at phylum level of HL and DL's intestinal microorganism in normal. The fecal simple from 6 health kids (HL) and 3 diarrhea kids (DL) were collected at day 0. Bacterial 16S rRNA genes were amplified and sequenced.

Table. S1 The 20 most predominant genus in the feces of kids with diarrhea (DL) and healthy kids (HL) at day 0..

Taxa	Genus	DL（%）	HL（%）	P Value
Bacteroidetes	Bacteroides	21.42±15.66	5.77±4.46	0.085162
	Parabacteroides	2.58±1.95	1.08±0.79	0.191844
	Alistipes	1.87±1.53	0.92±0.50	0.263596
Actinobacteria	Bifidobacterium	12.06±16.86	0.18±0.10	0.171904
	Collinsella	0.81±0.32	7.89±7.98	0.217591
	Paraeggerthella	0.27±0.25	0.57±0.74	0.567657
Verrucomicrobia	Akkermansia	8.34±8.14	0.25±0.13	0.068815
Firmicutes	Lactobacillus	5.38±0.73	18.51±9.39	0.070384
	Faecalibacterium	4.05±4.44	1.00±0.50	0.185458
	Anaerostipes	1.35±0.42	3.11±3.91	0.513774
	Ruminococcaceae_UCG-005	1.33±0.79	3.00±1.96	0.249556
	Erysipelotrichaceae_UCG-003	1.14±0.87	0.23±0.21	0.072702
	Tyzzerella	0.82±0.96	0.21±0.12	0.213658
	Phascolarctobacterium	0.74±0.75	4.07±6.31	0.448114
	[Ruminococcus]_torques_group	0.72±0.61	2.52±3.30	0.435862
	Clostridium_sensu_stricto_1	0.71±0.87	0.14±0.08	0.201146
Proteobacteria	Desulfovibrio	2.89±2.80	1.48±0.68	0.338315
	Campylobacter	2.22±2.93	0.33±0.30	0.209458
	Succinivibrio	0.44±0.45	0.89±1.05	0.547516
Euryarchaeota	Methanobrevibacter	0.22±0.11	1.61±1.35	0.157915

Data are means ± SEM. The significance was determined by Student’s t test.by Student’s t test.

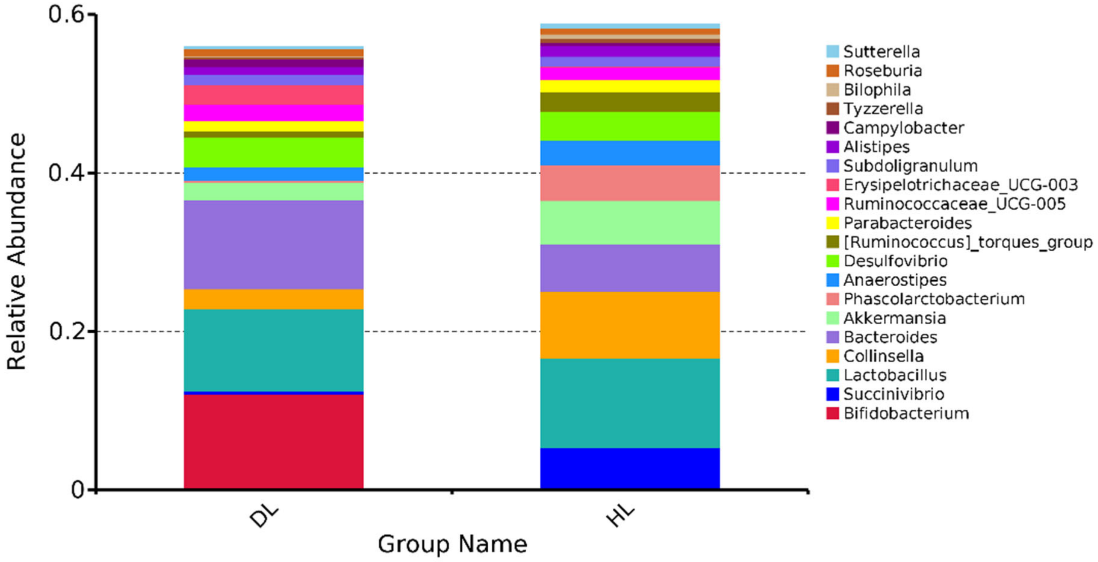

Figure S4 The relative abundance analysis at phylum level of HL and DL's intestinal microorganism treated with GBacillus-9. The fecal simple from 5 health kids (HL) and 3 diarrhea kids (DL) were collected at day 15. Bacterial 16S rRNA genes were amplified and sequenced.

Table. S2 Comparison of top 20 genus in the feces of the DLs and the HLs fed with Bacillus amyloliquefaciens-9 at day 15.

Taxa	Genus	DL（%）	HL（%）	P Value
Bacteroidetes	Bacteroides	11.17±6.00	5.93±6.59	0.341075
	Parabacteroides	1.28±0.57	1.57±1.66	0.803026
	Alistipes	0.95±0.51	1.36±1.23	0.644143
	Bilophila	0.18±0.06	0.53±0.66	0.451039
Actinobacteria	Bifidobacterium	12.07±16.85	0.17±0.10	0.171073
	Collinsella	2.51±1.01	8.33±8.41	0.327096
Verrucomicrobia	Akkermansia	2.16±2.17	5.43±7.69	0.544995
Firmicutes	Lactobacillus	10.36±2.90	11.17±7.61	0.878514
	Anaerostipes	1.69±0.44	3.11±3.90	0.597945
	[Ruminococcus]_torques_group	0.79±0.33	2.47±3.29	0.463954
	Erysipelotrichaceae_UCG-003	2.45±1.52	0.14±0.05	0.013655
	Ruminococcaceae_UCG-005	2.05±1.75	1.51±1.19	0.644583
	Tyzzerella	0.23±0.16	0.49±0.75	0.624537
	Phascolarctobacterium	0.27±0.15	4.46±6.11	0.32925
	Subdoligranulum	1.36±1.12	1.20±1.221	0.87309
	Roseburia	0.85±0.42	0.71±0.72	0.788879
Proteobacteria	Desulfovibrio	3.73±2.17	3.50±3.78	0.933392
	Campylobacter	0.95±1.07	0.42±0.27	0.35345
	Succinivibrio	0.42±0.46	5.10±8.87	0.447475
Euryarchaeota	Methanobrevibacter	0.38±0.26	1.12±0.95	0.282896

Data are means ± SEM. The significance was determined by Student’s t test.by Student’s t test.
